# Detection of Volatile Constituents from Food Lures by Tephritid Fruit Flies

**DOI:** 10.3390/insects9030119

**Published:** 2018-09-14

**Authors:** Tibebe Dejene Biasazin, Haimanot Teklemariam Chernet, Sebastian Larsson Herrera, Marie Bengtsson, Miriam Frida Karlsson, Joelle Kristin Lemmen-Lechelt, Teun Dekker

**Affiliations:** 1 Unit of Chemical Ecology, Department of Plant Protection Biology, Swedish University of Agricultural Sciences, P.O. Box 102, 230 53 Alnarp, Sweden; sebastianlh29@gmail.com (S.L.H.); marie.bengtsson@slu.se (M.B.); miriam.karlsson@slu.se (M.F.K.); joelle.kristin.lechelt@slu.se (J.K.L.-L.); teun.dekker@slu.se (T.D.); 2 Department of Zoological Sciences, Addis Ababa University, P.O. Box 1176, Addis Ababa, Ethiopia; himibabe@yahoo.com; 3 International Institute of Tropical Agriculture (IITA), Tri Postal, Cotonou 08 BP 0932, Benin

**Keywords:** *Bactrocera dorsalis*, behavior, electrophysiology, food-baits, olfactometer, Tephritidae

## Abstract

Tephritid fruit flies require protein for sexual and gonotrophic development. Food-based lures are therefore widely used in strategies to detect and control fruit flies in the Tephritidae family. However, these baits are attractive to a broad range of insect species. We therefore sought to identify volatiles detected by the fly antennae, with the goal to compose lures that more specifically target tephritids. Using gas chromatography-coupled electroantennographic detection (GC-EAD) we screened for antennal responses of four important tephritid species to volatile compounds from five commercially available protein-based baits. Antennal active compounds were reconstituted in synthetic blends for each species and used in behavioral assays. These species-based blends were attractive in olfactometer experiments, as was a blend composed of all antennally active compounds from all the four species we observed (tested only in *Bactrocera dorsalis*, Hendel). Pilot field tests indicate that the blends need to be further evaluated and optimized under field conditions.

## 1. Introduction

Fruit flies in the family Tephritidae are among the most economically important pests worldwide, and are a major threat to fruit production in Africa. Some of these fruit flies have been introduced outside their region of origin and become invasive pests [1]. *Bactrocera dorsalis*, *Bactrocera zonata* (Saunders), and *Zeugodacus cucurbitae* (Coquillett) have been introduced to Africa [2,3,4]. Following introduction, *B. dorsalis* has quickly dispersed throughout the region, causing unprecedented direct and indirect damages, including crop losses, quarantine restrictions and competitive displacement of native fruit flies such as *Ceratitis cosyra* (Walker) [5].

The host range of tephritid flies varies with species, some being polyphagous, such as *B. dorsalis*, *B. zonata* and *Ceratitis capitata* (Wiedemann), whereas others are oligophagous (*Z. cucurbitae*) or even monophagous (*Bactrocera oleae*, Rossi) [6]. In spite of their diverse oviposition substrates, however, all adult tephritid species need proteinaceous substrates for sexual maturation and development of ovaries [7,8]. Adult flies, particularly females, extensively forage for proteinaceous food sources [9]. Various food-baits have been developed and are being used as a main component of female-biased attractants [10]. Food-baits offer substantial advantages, as they attract both males and females of multiple species [11]. An important alternative to a food-bait for monitoring or control is male annihilation. This uses male lures, often called parapheromones [12], which, although highly attractive to males and species specific, seems inadequate for African smallholder settings as the small farm size causes a steady influx of dispersing flies from surrounding plots [13]. In addition, the relatively high specificity seems a disadvantage when a diverse guild of fruit flies co-occurs on a crop. Suppression of a single species may result in a competitive increase and replacement of the main pest species by another [5]. In contrast, synthetic lures derived from food volatiles that are universally detected across sexes and species, may be used in simultaneously suppressing multiple species and target population control more directly by removing females.

Volatiles from food sources that attract tephritid fruit flies are largely byproducts of microbial metabolic pathways, lysis and protein breakdown, and serve as important indicators of resources critically needed for tephritid reproductive success [14,15]. Accordingly, the life history of tephritids is closely associated with microorganisms. Microbes are a source of protein, minerals and vitamins, and contribute to fitness and reproductive success [16,17]. In addition, microbes take part in the host interaction of larval and adult fruit flies by either detoxifying toxic substances during feeding or by degrading or modifying oviposition substrates [18]. Fruit flies inoculate microorganisms from one fruit to another in order to facilitate oviposition and larval development [19]. Many bacteria associated with fruit flies are also capable of fixing atmospheric nitrogen into usable forms such as ammonia [17], which may be important as fruits are generally low in protein content.

Identification of volatile constituents of food lures include protein-signifying compounds such as nitrogen-containing compounds, some of which are important fruit fly attractants [20,21]. Nitrogen-free volatiles are also produced and important for the attraction of fruit flies to protein sources [11]. A three-component synthetic lure containing putrescine, trimethylamine and ammonium acetate, Biolure^®^, is a good example of how such research can lead to new odor-based tools for fruit fly monitoring and control [22,23,24,25,26,27]. However, none of the efforts have used the fly’s sensory organs to identify which compounds in the fermentation and protein hydrolysate baits are actually detected by the antennae.

In the current study, we used gas chromatography-coupled electroantennographic detection (GC-EAD) together with gas chromatography-coupled mass spectrometer (GC-MS) to identify antenally active volatiles from five commercially available food-baits. We compared antennal responses from three polyphagous tephritid pests *B. dorsalis*, *B. zonata,* and *C. capitata* and one oligophagous pest *Z. cucurbitae*, to volatiles of the five lures. Furthermore, we compared the antennal responses with an existing database of responses of olfactory receptors of the non-pest fruit fly *Drosophila melanogaster* (DoOR). Based on antennal responses of the four tephritid fruit flies, we constructed one general blend, and based on antennal responses of each fruit fly species we constructed species-based blends and tested these in an olfactometer assay and in the field.

## 2. Materials and Methods

### 2.1. Insects

Pupae of four fruit fly species (*B. dorsalis*, *B. zonata*, *C. capitata* and *Z. cucurbitae*) were provided by the International Atomic Energy Agency (IAEA, Vienna, Austria), from where colonies were started. Flies were kept at 25–29 °C, 60–65% RH and 12 h:12 h light:dark photoperiod. Larvae were provided with a carrot-based diet consisting of carrot powder (Sela, Aulendorf, Germany), sucrose (Nordic Sugar AB, Malmö, Sweden), baker’s yeast (Jästbolaget AB, Sollentuna, Sweden), wheat bran (Kungsörnen, Stockholm, Sweden), nipagin (methyl 4-hydroxybenzoate, Merck, St. Louis, MO, USA), and linolenic acid (Merck) with 121:80:12:10:3:1 grams of proportions respectively in 100 mL of water. Adults were fed with a mixture of 3:1 sugar:yeast, and were provided with water-soaked cotton balls on a 9 cm plastic petri dish. Adults were kept in polyester netting bugdorm-430430 cages (L32.5 × W32.5 × H32.5 cm) with mesh sizes (96 × 26/680 µm) opening. Flies were reared continuously for 6 generations. Unmated females (4–5 days old) were used for all experiments.

### 2.2. Commercial Baits

Five commercially available protein and fermentation-based attractive baits were chosen for this study:

1. brewer’s yeast waste (St. George brewery, Addis Ababa, Ethiopia), is a byproduct from the brewing process that is normally tossed off. It is rich in yeast and has been exploited as an alternative source of protein for use in tephritid fruit fly management [28];
2. baker’s yeast (*Saccharomyces cervisiae*) (Jästbolaget AB, Sollentuna, Sweden), activated with sugar, is a common diet for adult tephritid fruit flies. It is also an essential component of tephritid larval media (see Section 2.1);
3. GF-120 success bait (Dow AgroSciences, Indianapolis, IN, USA), is a combination of spinosad, enzymatically hydrolyzed protein, sugars, adjuvants, and conditoners that attracts fruit flies [29];
4. Anamed (ISCA Technologies, Riverside, CA, USA), is a protein-based attractant that targets a wide range of tephritid fruit flies;
5. Torula yeast (*Candida utilis*) (ISCA Technologies, Riverside, CA, USA), is a species of yeast which is often grown on wood sugars left over after paper production. These baits were chosen as representatives of the different types of attractive tephritid baits used by growers [28,29,30,31].

### 2.3. Volatile Collection and GC-MS

Volatiles were collected from aqueous solutions of each bait. The brewer’s waste was in liquid form and used directly. The baker’s yeast (12 g) was combined with 4 g of sugar and mixed in 150 mL of water and left to ferment for 24 h. The three commercial food baits were mixed with water according to the instructions by the companies for field use. Sampling of the volatile components was made by enclosing 50 mL of each of the aqueous solutions of protein baits in glass wash bottles (250 mL, Lenz Laborglas GmbH & co. KG, Wertheim, Germany). Charcoal-purified air entered the system from the air pushing section of a pump (12 V, KNF-Neuberger, Freiburg, Germany) at a rate of 0.5 L min^−1^. The sucking section of the pump was connected to columns (55 mm, id = 3 mm) filled with 50 mg of Porapak Q (80–100 mesh) to adsorb the volatiles emitted from the samples. Aeration was run for 5 h, and then columns were rinsed with 500 µL redistilled hexane (LabScan, Malmö, Sweden). All samples were first individually analyzed with GC-MS, then combined and re-analyzed. The pooled headspace samples were used in electrophysiological and behavioral experiments.

### 2.4. Electrophysiology

Flies were immobilized in a 200 μL micropipette tip with the antennae protruding from its narrow aperture. Capillaries filled with Beadle–Ephrussi ringer solutions (7.5 g NaCl, 0.35 g KCl, 0.29 g CaCl_2_ dissolved in 1 L of distilled water) were used to create conduction between the electrodes and one insect antenna. The glass capillary attached to the reference electrode was inserted into the head of the fly, and the glass capillary on the recording electrode was connected to the tip of the antennae. The recording electrode was connected to a pre-amplifier probe and then to a high impedance GC amplifier interface box (IDAC-2; Syntech, Kirchzarten, Germany). Aliquots of 2 μL of the headspace sample were injected into a GC. The temperature program of the GC oven was set to start at 40 °C and held for 3 min, increasing by 10 °C min^−1^ to 280 °C and held for 8 min. The inlet was in splitless mode with temperature of 250 °C, pressure of 12.96 psi and total flow of 38.2 mL min^−1^. The sample was carried by H_2_ through an HP-5MS, 5% Phenyl Methyl Siloxane capillary column (Agilent, 30.0 m × 250 μm × 0.25 μm nominal). The effluent from the GC was split 1:1 between the flame ionization detector (FID) and the antenna. The capillary column for the EAD passed from a Gerstel (Mühlheim, Germany) olfactory detection port-2 transfer line tracking the GC oven temperature into a glass tube (30 cm × 8 mm), where it was mixed with charcoal filtered and humidified air at a flow rate of 1.5 L min^−1^ and passed over the fly’s antenna. EAD peaks were analyzed using Syntech data acquisition system software (GC-EAD 2012 v1.2.4, Syntech, Kirchzarten, Germany).

### 2.5. Chemical Analysis

Simultaneous FID-EAD peaks from the headspace sample were identified by injecting 2 μL of the extract on a combined gas chromatograph and mass spectrometer (GC-MS; 6890 GC and 5975 MS; Agilent Technologies Inc., Santa Clara, CA, USA), operated in the electron impact ionization mode at 70 eV. The GC was fitted with an HP-5MS Ultra Inert fused capillary column (60 m × 0.25 mm i.d., 0.25 μm film thickness, J&W Scientific, Folsom, CA, USA). The GC oven program was the same as that of the GC-EAD program. Helium was the carrier gas at a constant flow mode of 1.8 mL min^−1^, the average velocity was 35 cm s^−1^. Mass spectra were scanned from *m*/*z* 30–300 and acquired data were collected and analyzed using HP Chemstation software with Kovat’s indices and mass spectra from our own library (Alnarp 11) and NIST 05 database libraries and confirmed by injection of authentic standards: 3-methyl-1-butanol ≥98%, ethyl butanoate ≥98%, butyl acetate ≥99%, 2-methylpyrazine ≥99%, 3-methylbutyl acetate ≥95%, 2,5-dimethylpyrazine ≥99%, 2,6-dimethylpyrazine ≥98%, ethyl (*E*)-2-methylbut-2-enoate ≥98%, benzaldehyde ≥99.5%, β-myrcene ≥95%, ethyl hexanoate ≥99%, 2-methoxyphenol ≥99%, 1-phenylethyl acetate >98%. All compounds were purchased from Merck. For quantification, 100 ng of heptyl acetate (99.8%; Merck) was added as an internal standard.

### 2.6. Synthetic Blends

Based on the GC-EAD results of each of the four fruit fly species and the relative quantities found in the headspace (Table 1), five synthetic blends were formulated in paraffin oil (Merck). One blend was composed of all 13 compounds that elicited variable antennal responses in any of the fruit fly species, named “13-blend”. The other four blends were formulated based on the response profiles of each of tephritid fruit fly species under study (*B. dorsalis*, *B. zonata*, *C. capitata* and *Z. cucurbitae*) and named accordingly (*dorsablend*, *zonablend*, *capiblend* and *cucublend*) (Table 1). The blends were tested in a behavioral olfactometer assay, see below. The odor blend was applied on a filter paper (Whatman™ Grade 1). The release rate of the compounds from the filter paper was quantified for each of the six synthetic blends from the filter paper sources and adjusted to match the ratios in the headspace using solid-phase micro extraction (SPME) on a fiber coated with (DVB/CAR/PDMS 50/30 μm; Supelco, Bellefonte, PA, USA) and GC-MS analysis.

### 2.7. Olfactometer Experiments

**Figure 1:**
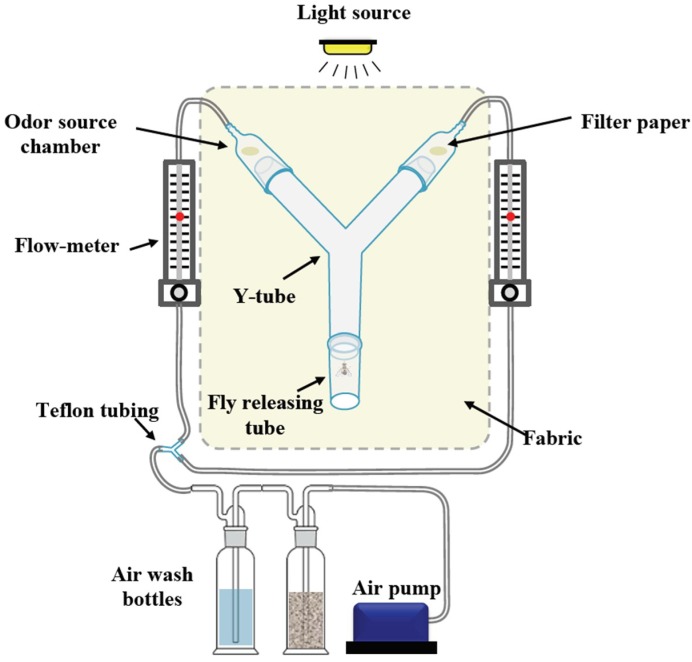
Schematic design of the Y-tube apparatus used in the 2-choice olfactometer experiments. The Y-tube bioassay apparatus was placed in a box made from white fabric to disperse the light more evenly and to avoid disturbances induced by movement. Pumped air was cleaned in a series of bottles with activated charcoal and distilled water. An airflow of 0.5 L min^−1^, adjusted using two flowmeters, reached via Teflon tubing, the glass odor source chambers at each side of the upstream tube. Treatments loaded on filter paper were placed inside the odor source chambers. Flies were released individually at the entrance of the Y-tube common arm, and were considered to have made a choice when the female reached the odor source chamber.

Two-choice olfactometer experiments were conducted in a Y-tube bioassay apparatus, placed in a box made of white fabric (40 cm × 40 cm × 50 cm) to disperse light more evenly and to avoid disturbances by movements of the experimenter (Figure 1). The Y-tube was made of a borosilicate glass tube with an id of 3.1 cm. The length of the upstream arms was 16 cm each and the common arm was 14 cm long. The upstream end consisted of glass odor source chambers (8.5 cm and 2 cm id) containing a stimulus (10 µL) on a Whatman^TM^, grade 1, circular filter paper (20 mm diam.). An air flow was created using an Elite 802 pump (Rolf C. Hagen U.K. Ltd., Castleford, UK), pumped through a teflon tubing and passed through series of glass wash bottles for activated charcoal filtering and humidification. The air leaving the wash bottles was split into two teflon tubings, each adjusted using a flow meter (BA-4AR, Kytola instruments, Muurame, Finland, Figure 1), and then entering the glass odor source chamber with an airstream of 0.5 L min^−1^. Experimental flies were starved for 5 h prior to experiments; individual flies were transferred to vials and moved to a climate chamber (25 °C, 65% RH) 1 h prior to experiment to acclimatize flies.

Behavioral experiments assessed attraction of female *B. dorsalis* to 1. a mix of five commercial baits in equal ratios (mix of baits) 2. the pooled headspace extract of the five lures, 3. the 13-component synthetic blend and 4. the *B. dorsalis*-based synthetic blend (dorsablend). Following these experiments, female *B. zonata*, *C. capitata* and *Z. cucurbitae* were tested towards zonablend, capiblend and cucublend respectively (Table 1). Flies were released individually at the entrance of the Y-tube’s common arm, and were considered to have made a choice when the female reached the odor source chamber (Figure 1). Flies that did not enter the odor source chamber within 10 min were considered non-responders. The positions of the stimuli were rotated and odor sources were replaced with new stimuli on clean filter paper after every five flies. Forty flies were released for each experiment. Glass tubes were cleaned and rinsed with 70% ethanol and dried in an oven (350 °C) for 8 h.

### 2.8. Field Experiments

Field experiments were conducted from April to June 2016, in mixed-fruit orchards belonging to smallholder farmers of Chano-Mille, Arbaminch, Southern Ethiopia. Traps used for this experiment were Easytrap^®^ (14 × 9 × 5 cm J.P. Ros, INIA, Madrid, Spain), which consists of a two-port rectangular plastic with an inbuilt hook and a capacity of holding up to 400 mL of liquid attractants. A synthetic blend in paraffin oil based on the response of female *B. dorsalis* (dorsablend) was compared to GF-120 and Torula yeast. Three plots (~70 m × 70 m) were selected for experiments. Traps containing 200 mL of the commercial baits (GF-120 and Torula yeast) and traps containing 2 mL of the dorsablend and paraffin oil were hung at every corner of each plot. The dorsablend and the paraffin oil were applied on a cotton wick in a 4 mL vial and placed inside the trap. Distances between traps were 30 m. Traps were checked and serviced every week, loaded with fresh lures, and rotated. The experiment was replicated four times, with each replicate being one week of trap-catches.

### 2.9. Statistical Analysis

Attraction of female fruit flies to odor sources in an olfactometer was analyzed with a Chi Square test goodness-of-fit to determine if the response differed from a 50:50 distribution of flies. General linear model (GLM) with a poisson family followed by Tukey HSD post hoc test was used to analyze the field data. Three replicates of antennal responses of all fruit fly species were used for calculating the average relative response of EAD amplitudes. First, responses in each EAD trace were normalized by dividing the depolarization of each individual response by a weighted mean of all responses in that trace. This weighted mean was calculated as the back transformed (exp) average of the ln transformed depolarization values of all responses in that trace. Normalization thus makes the response relative, and prevents confounding the average by differences in absolute sensitivity of the antennal preparation. The transformation on the other hand makes the weighted average less sensitive to outliers. After this, the normalized responses were averaged across traces and scaled on a scale from 0 to 1 by dividing it by the total sum of average normalized responses, and used for tile plotting using ggplot 2 [32]. To simulate the antennal response of *D. melanogaster*, the DoOR response strength of a given receptor to a compound [33] was multiplied by the number of receptor neurons expressing that receptor in the antennae [34], summed across responding receptors, and weighed. Data analysis was performed with R (version 3.3.2) [35].

## 3. Results

### 3.1. GC-EAD and Volatile Identification

**Figure 2:**
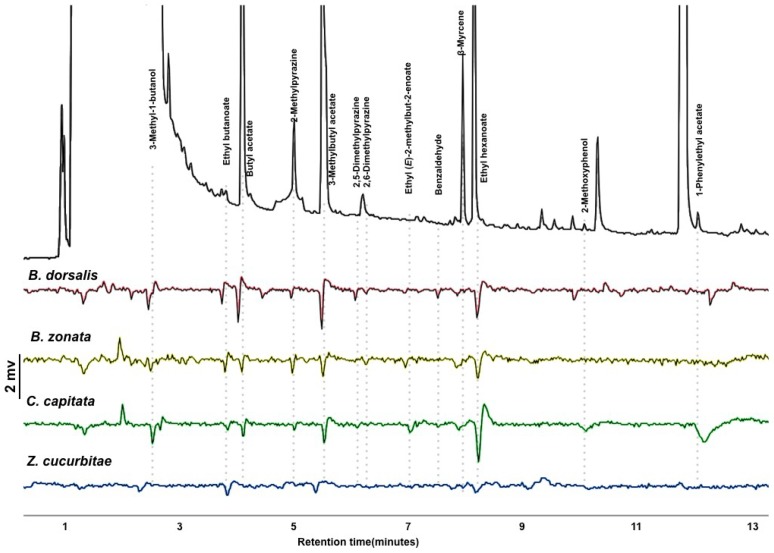
Gas chromatograph-coupled electroantennogram detection response of the four tephritid pest species tested to the headspace extract of the mixture of food-baits. Top trace (black): GC-FID trace of the headspace of the mix of bait; bottom 4 traces (colored): EAD traces for the four fruit fly species tested.

GC-EAD analysis revealed 14 active compounds that elicited antennal responses from any of the four species tested (Figure 2). All species responded to 3-methylbutyl acetate and ethyl hexanoate. *Bactrocera dorsalis* responded to nine compounds, *B. zonata* to ten compounds, *C. capitata* to eleven compounds, and *Z. cucurbitae* to six compounds. The three polyphagous pests (*B. dorsalis*, *B. zonata* and *C. capitata*) shared the detection of 7 compounds. The identity of one compound that appears at the beginning of the solvent peak was unconfirmed. Thirteen of the compounds were identified and verified (Figure 2). These were used to formulate the synthetic blends outlined in Table 1, and used in subsequent olfactometer and field experiments.

### 3.2. Antennal Sensitivity and DoOR Comparison

**Figure 3:**
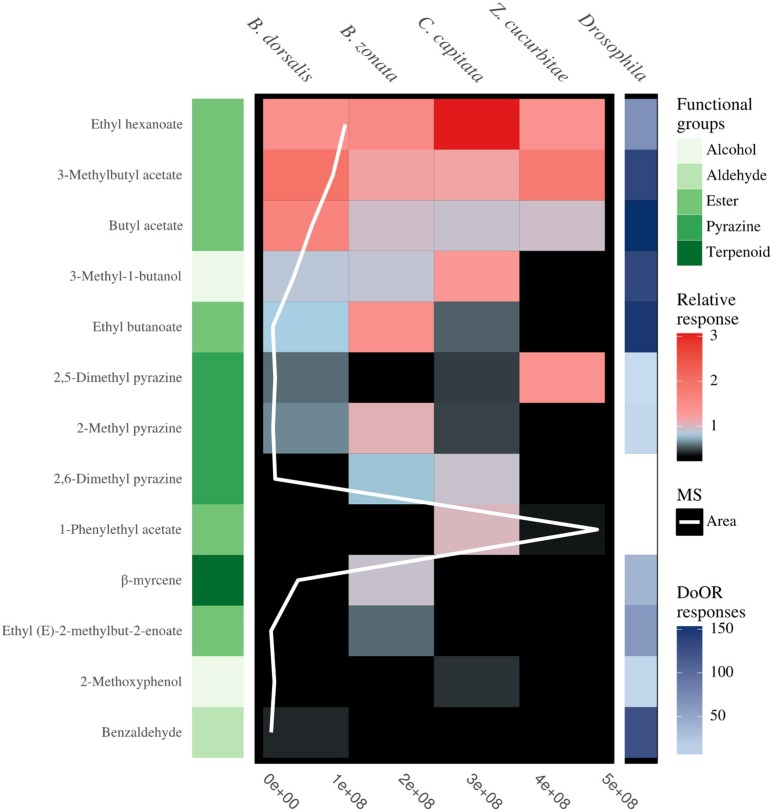
The above plot from left to right is composed of (1) Compound name (2) the functional classes of the compounds (3) antennal sensitivity of the four fruit fly species (*B. dorsalis*, *B. zonata*, *C. capitata* and *Z. cucurbitae*) to compounds in food baits with the MS area (the sum of ions) (4) simulated olfactory response of *D. melanogaster* to the compounds. Compounds from five chemical groups were identified to induce an antennal response and include, terpene, pyrazines, esters, aldehyde and alcohols (from dark green to light green respectively). The average relative sensitivity of the fly’s antennae ranges from black (no response) to red (6× averaged EAD response). The olfactory receptors response of *D. melanogaster* increases from light blue to dark blue, with white tile indicating compounds not present in the DoOR database.

The thirteen compounds detected by fruit flies belonged to five different functional groups, including six esters, three pyrazines, two alcohols, one aldehyde and one terpene (Figure 3). Comparison with the DoOR database revealed that 11 of the 13 compounds are detected by olfactory receptors of *D. melanogaster* with varying sensitivities (Figure 3). 2,6-dimethylpyrazine has not been reported to induce a response in *D. melanogaster*, while α-methylbenzyl acetate is not present in the DoOR database.

### 3.3. Olfactometer Experiments

#### 3.3.1. Behavioral Response of *B. dorsalis*

**Figure 4:**
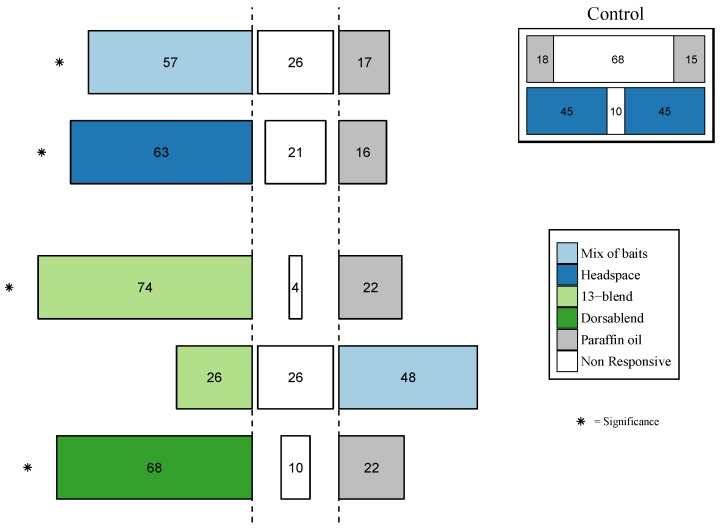
Choice of *B. dorsalis* females between treatments (mix of baits, headspace, 13 blend and dorsablend) and control (paraffin oil, or mix of bait), in a y-tube olfactometer. Mix of baits, is a mixture of brewer’s yeast waste, baker’s yeast, torula yeast, anamed & GF-120. Top right inset: experiments consisting of (1) headspace against headspace and (2) paraffin oil against paraffin oil. Forty flies were released individually and the percentage of flies that made a choice is presented within the bars, middle bars (white) represent non-responding flies. α = 0.05, * *p* < 0.05.

In olfactometer assays with food-bait odors, female *B. dorsalis* were highly attracted to the mixture of baits made from five food-bait lures (χ^2^ (1) = 4.764, *p* = 0.02), the pooled headspace sample from these products (χ^2^ (1) = 18.13, *p* < 0.05), the synthetic 13-blend (χ^2^ (1) = 6.545, *p* = 0.01) and the species-specific “Dorsablend” (blend based on responses of *B. dorsalis*) (χ^2^ (1) = 9, *p* = 0.002) compared to the paraffin oil control (Figure 4). Female *B. dorsalis* did not show a preference for the mixture of baits and the synthetic 13-blend (χ^2^ (1) = 1.47, *p* = 0.22), (Figure 4). Control trials with paraffin oil or pooled headspace in both odor source chambers showed that there was no directional bias in the olfactometer set-up with *B. dorsalis* (Figure 4 inset on top right).

#### 3.3.2. Behavioral Response of other Tephritid Species

**Figure 5:**
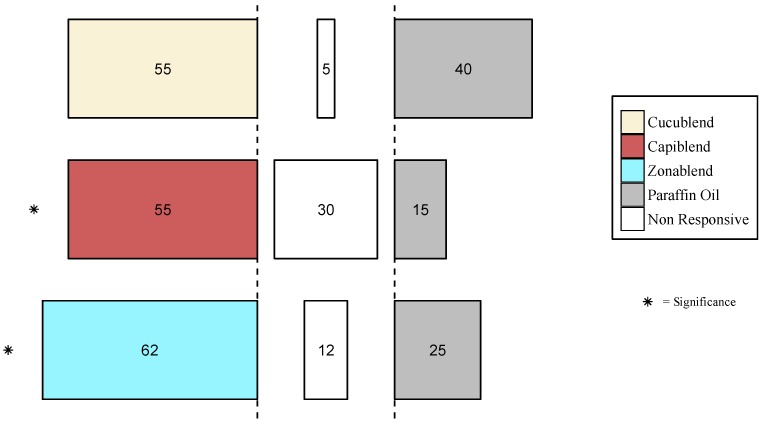
Behavioral response of *Z. cucurbitae*, *C. capitata* and *B. zonata* to cucublend, capiblend and zonablend. Forty flies were released individually and the percentage of flies that made a choice is presented within the bars. Colored bars indicate synthetic blends, gray bars indicate control (paraffin oil) and middle bars (white) represent non-responsive flies. α = 0.05, * *p* < 0.05.

Synthetic blends based on each species’ antennal responses were attractive for female *B. zonata* (zonablend, χ^2^ (1) = 6.42, *p* = 0.01), and female *C. capitata* (capiblend, χ^2^ (1) = 9.14, *p* = 0.002), but not for female *Z. cucurbitae* (cucublend, χ^2^ (1) = 0.947, *p* = 0.330) (Figure 5).

### 3.4. Field Experiment

#### Trap Catches of *B. dorsalis* with Protein-Based Baits

**Figure 6:**
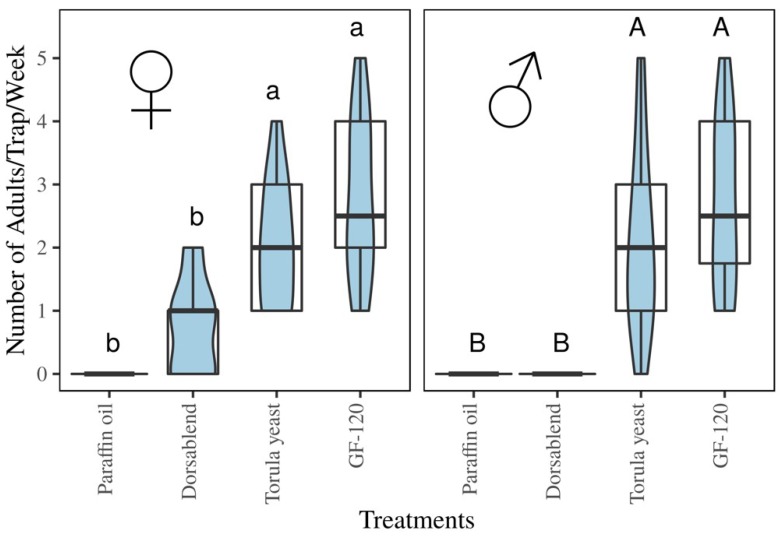
Female and male *B. dorsalis* fruit flies caught per trap in commercial lures (GF-120 & torula yeast) compared to the synthetic dorsablend. Different letters indicate significant differences. Boxplot shows the spread and the violin plot shows density of flies per trap.

In the field in Arbaminch, Ethiopia, traps caught roughly equal numbers of males and females F (1,24) = 1.154, *p* = 0.293 (Figure 6). Traps with the commercially available protein-based baits (GF-120 and Torula yeast) caught significantly higher numbers of flies compared to the dorsablend (Tukey: *p* < 0.05) (Figure 6). Although the attractiveness of the synthetic dorsablend was not significantly different from the paraffin control for either sex, it only trapped female flies. Even though trap entry was low, female flies were frequently observed landing on the trap without entering. In a separate study, an experiment with male-specific lures conducted in the same area around the same period, male *B. dorsalis* flies were caught in the order of hundreds per day per trap [13], indicating that flies were abundant in the area.

## 4. Discussion

In the current study, our aim was to identify key compounds that attract fruit flies to feeding sites, with a view to develop synthetic lures that are more specific than the currently available broad-spectrum protein baits. We have identified a total of 13 physiologically active compounds from food-based fermentation products that are detected by the four species in our study. Most compounds identified in this study are signature compounds for microbial activity and protein breakdown products. As microorganisms constitute important resources, supplying the much-needed proteins, their detection through associated volatiles induce strong behavioral responses in Tephritidae. The majority of the compounds identified here are also ligands of *D. melanogaster* olfactory receptors (see heatmap Figure 3, [33]), a species that shares the strong association with microbes, which underlines their importance in signaling fermentation and protein sources [36,37,38,39,40].

Pyrazines are heterocyclic aromatic volatile compounds associated with gut bacteria of fruit flies and other insects. The importance of pyrazines in insect-microbe-plant interactions is noted in other contexts. *Drakaea glyptodon* (Orchidaceae) flowers emit pyrazines that are also found as pheromone of the female wasp *Zaspilothynnus trilobatus* (Hymenoptera: Thynninae) pheromone, to attract males for pollination [41]. Different forms of pyrazines have been reported as components of tephritid male sex pheromones [42,43,44]. Three of the compounds identified from the headspace extracts of the protein-based baits in the current study are pyrazines, including 2-methylpyrazine, 2,5-dimethylpyrazine and 2,6-dimethylpyrazine. 2-methylpyrazine has been isolated from several bacterial filtrates including *Klebsiella pneumoniae* and *Citrobacter freundii* and its attractiveness to the fruit fly *A. ludens* was evaluated singly, but without success [38]. In the current study, 2-methylpyrazine was detected by the antennae of the three polyphagous pests, *B. dorsalis*, *B. zonata* and *C. capitata*. It was also part of the specific blends which elicited a behavioral response in the olfactometer for each of these species. Although, 2-methylpyrazine did not induce a response in the antennae of female *Z. cucurbitae*, it is reported as a male rectal gland component of *Z. cucurbitae*, and suggested to have a role in sexual communication [42]. The lack of response in the current study may be either due to the below threshold amounts in the sample, or that this compound is detected by another olfactory organ, such as the maxillary palp.

Several organic volatile compounds from food-based fermentation products identified in this study are products of microbial modifications of fatty acids, aromatic amino acids and carbohydrates [45]. Epsky et al. [46], identified 3-methyl-1-butanol from *Enterobacter agglomerans* as a main component attracting *Anastrepha suspensa*. This substance was detected as a minor component in corn protein hydrolyzate bait [47], and also from fermented host fruit of *A. ludens* [38,48]. However, the compound is not unique to fermentation products, as it is also a main component of several fruits and elicits responses in other fruit flies such as the spotted wing *Drosophila suzukii* [49]. In our study, 3-methyl-1-butanol was part of the dorsablend, zonablend and capiblend, but not part of the cucublend, which may be a reason why this blend was not attractive to *Z. cucurbitae*.

A main limitation of utilizing the general proteinaceous or fermentation baits for control of tephritid flies is that they are not target-specific [50]. Many other non-target insect species are attracted to these sources for food, which may negatively impact their population [24,25,51]. Interestingly, in our study each species has a unique response profile to these compounds, which was unanticipated, as in drosophilid fruit fly species, much of antennal circuitry appeared to be conserved [52] and all tephritid species tested here rely on protein sources for maturation. This we used for the development of synthetic blends for each tephritid species, which were attractive for *B. dorsalis*, *B. zonata* and *C. capitata*. There is an opportunity to further develop these blends to target the individual species, or to develop a more broadly detected blend that could be used for several species concurrently. Adjusting blends to differences or commonalities in antennal sensitivity between species reported here, may allow for further developing lures that are selectively target few or many tephritid species.

## 5. Future Research

The translation of our laboratory findings to the field needs further research. Whereas the dorsablend was very attractive in the laboratory, field catches of the dorsablend trap were low although selectively attracting *B. dorsalis*. Commercial baits were also more attractive. However, female *B. dorsalis* flies were frequently observed on and around the dorsablend trap without entering the trap (T.D.B., personal observation). The lack of trap entry could be due to low amounts of volatiles in our blends, the short field life of these lures compared to the control, and the inadequate control of release rates and ratios over time. It may also indicate that some important compounds detected at short range are missing in the dorsablend or in the trap. These include water [53,54], as well as several nitrogenous compounds such as ammonia and several amines known as tephritid attractants [27,55], but not detected in our GC-EAD. Generally, identification and quantification of primary amines by GC can be problematic [56]. In addition, detection of these compounds in insects may be dominated through gustatory and ionotropic receptors rather than olfactory receptors [57,58]. Receptor neurons that house ionotropic receptors are typically very small, and responses are often weak, which may affect detectability. Similarly, acetic acid is also detected by coeloconic sensilla and have gone unnoticed in our GC-EAD recordings [59]. Further studies should combine known attractants with the compounds identified here to enhance trap catch and/or the specificity of tephritid lure.

## Figures and Tables

**Table 1:** Chemical components of the four species-based and one general blend, identified by GC-EAD and GC-MS.

Blend Components (General Blend)	Ratio (ng µL^−1^)	CAS	Kovats Index	*B. dorsalis* dorsablend	*B. zonata* zonablend	*C. capitate* capiblend	*Z. cucurbitae* cucublend
3-Methyl-1-butanol	35	123-51-3	763	*	*	*	
Ethyl butanoate	3	105-54-4	810	*	*	*	
Butyl acetate	62	123-86-4	821	*	*	*	*
2-Methylpyrazine	4	109-08-0	831	*	*	*	
3-Methylbutyl acetate	94	123-92-2	877	*	*	*	*
2,5-Dimethylpyrazine	7	123-32-0	911	*		*	*
Benzaldehyde	1	100-52-7	963	*			
Ethyl hexanoate	112	123-66-0	993	*	*	*	*
2,6-Dimethylpyrazine	7	108-50-9	912		*	*	
Ethyl (E)-2-methylbut-2-enoate	1	5837-78-5	937		*		
β-Myrcene	41	123-35-3	987		*		*
2-Methoxyphenol	6	90-05-1	1082			*	
1-Phenylethyl acetate	249	93-92-5	1189			*	*

* blend composition.
